# Craniofacial dysmorphology in 22q11.2 deletion syndrome by 3D laser surface imaging and geometric morphometrics: Illuminating the developmental relationship to risk for psychosis

**DOI:** 10.1002/ajmg.a.36893

**Published:** 2015-02-18

**Authors:** Sarah Prasad, Stanislav Katina, Robin J. Hennessy, Kieran C. Murphy, Adrian W. Bowman, John L. Waddington

**Affiliations:** ^1^ Department of PsychiatryRoyal College of Surgeons in IrelandBeaumont HospitalDublinIreland; ^2^ School of Mathematics and StatisticsUniversity of GlasgowGlasgowUK; ^3^ Institute of Mathematics and StatisticsMasaryk UniversityBrnoCzech Republic; ^4^ Molecular and Cellular TherapeuticsRoyal College of Surgeons in IrelandDublinIreland

**Keywords:** 22q11.2 deletion syndrome, velocardiofacial syndrome, schizophrenia, craniofacial dysmorphology, 3D laser surface imaging, geometric morphometrics

## Abstract

Persons with 22q11.2 deletion syndrome (22q11.2DS) are characterized inter alia by facial dysmorphology and greatly increased risk for psychotic illness. Recent studies indicate facial dysmorphology in adults with schizophrenia. This study evaluates the extent to which the facial dysmorphology of 22q11.2DS is similar to or different from that evident in schizophrenia. Twenty‐one 22q11.2DS‐sibling control pairs were assessed using 3D laser surface imaging. Geometric morphometrics was applied to 30 anatomical landmarks, 480 geometrically homologous semi‐landmarks on curves and 1720 semi‐landmarks interpolated on each 3D facial surface. Principal component (PC) analysis of overall shape space indicated PC2 to strongly distinguish 22q11.2DS from controls. Visualization of PC2 indicated 22q11.2DS and schizophrenia to be similar in terms of overall widening of the upper face, lateral displacement of the eyes/orbits, prominence of the cheeks, narrowing of the lower face, narrowing of nasal prominences and posterior displacement of the chin; they differed in terms of facial length (increased in 22q11.2DS, decreased in schizophrenia), mid‐face and nasal prominences (displaced upwards and outwards in 22q11.2DS, less prominent in schizophrenia); lips (more prominent in 22q11.2DS; less prominent in schizophrenia) and mouth (open mouth posture in 22q11.2DS; closed mouth posture in schizophrenia). These findings directly implicate dysmorphogenesis in a cerebral‐craniofacial domain that is common to 22q11.2DS and schizophrenia and which may repay further clinical and genetic interrogation in relation to the developmental origins of psychotic illness. © 2015 The Authors. *American Journal of Medical Genetics Part A* Published by Wiley Periodicals, Inc.

## INTRODUCTION

Chromosome 22q11.2 deletion syndrome (22q11.2DS), also known as velocardiofacial syndrome (VCFS) or DiGeorge syndrome, is the most frequently occurring chromosomal microdeletion syndrome in humans, with an estimated incidence of 1 in 4,000 live births [Goldberg et al., 1993; Robin and Shprintzen, 2005]. This syndrome comprises multiple abnormalities, with an extensive and variable phenotype with over 180 clinical features; common abnormalities include speech and palatal anomalies, cardiac outflow tract defects, immune disorders, learning difficulties, psychiatric disorders and a characteristic facial dysmorphology [Kobrynski and Sullivan, 2007; Shprintzen, 2008]. There is a large body of evidence to indicate an unequivocal association between 22q11.2DS and risk for psychotic illness, with approximately 25% of adults with 22q11.2DS developing psychosis [Murphy et al., 1999; Murphy, 2002; Bassett et al., 2005]. Anomalies in craniofacial and cardiac structures evident in 22q11.2DS, together with psychotic psychopathology, may reflect abnormal neural crest migration and subsequent mal‐development; thus, a gene [or genes] within the 22q11 region may be involved in neural crest migration and/or differentiation, such that haploinsufficiency of the gene(s) may disrupt development, leading to multiple tissue and organ anomalies [Walker and Trainor, 2006; Aggarwal and Morrow, 2008; Momma, 2010].

Early embryological developmental abnormalities may be involved in the etiology of psychosis, not only in 22q11.2DS but also in schizophrenia among the general population [Waddington et al., 2012]. The characteristic facial dysmorphism of 22q11.2DS has been previously described clinically, anthropometrically, and using two‐dimensional (2D) facial images [Butts, 2009]. However, none of these techniques captures and analyses facial dysmorphology in its inherent three‐dimensionality. Recently, three‐dimensional (3D) surface imaging has been applied to children with 22q11.2DS to capture facial dysmorphology, primarily in the context of statistical discrimination from other childhood developmental syndromes, rather than quantification and specification of 22q11.2DS dysmorphology itself [Hammond et al., 2004, 2005; Sinderberry et al., 2013].

We have recently applied 3D surface imaging and geometric morphometrics to quantify facial dysmorphology in adults with schizophrenia [Hennessy et al., 2007] and bipolar disorder [Hennessy et al., 2010]. The anterior brain and face evolve in embryological intimacy over early fetal life [Diewert et al., 1993; Marcucio et al., 2005, 2011] and the developmental biology of facial morphogenesis is better understood than brain morphogenesis. Therefore, detailed, quantitative assessment of facial dysmorphology in 22q.112DS, and the extent to which it is similar to or different from facial dysmorphology in schizophrenia, may lead to greater understanding of brain dysmorphogenesis in 22q11.2DS and its developmental relationship to psychosis in 22q11.2DS, schizophrenia and bipolar disorder. Described here is the first study to investigate facial dysmorphology in individuals with 22q11.2DS, compared to unaffected sibling controls, using the application of 3D laser surface imaging and geometric morphometric techniques similar to those applied previously to schizophrenia and bipolar disorder.

## MATERIALS AND METHODS

### Participants

Approval for this study was obtained from the Research Ethics Committees of Beaumont Hospital and Our Lady's Hospital for Sick Children, Dublin, Ireland, and Belfast City Hospital and the Office for Research Ethics Committees, Northern Ireland, UK; for patients aged 18 or above, written, informed consent to participation was obtained from the patient, while for patients aged under 18, written, informed permission to participate was obtained from a parent/guardian and assent obtained from the individual. Patients were recruited through the following sources: the National Center for Medical Genetics, Our Lady's Hospital for Sick Children, Dublin; the Northern Ireland Regional Genetics Center, Belfast; and two 22q11.2DS support groups (22q11.2DS Ireland and Max Appeal UK).

Patients were drawn from 45 individuals having genetically confirmed 22q11.2DS in the absence of any other chromosomal abnormality [20 males, 25 females; mean age 14.6 (SD 8.9)]; among these 45 individuals with 22q11.2DS, 35 were de novo deletions, and thus independent of each other, while 10 were familial, inherited deletions and related to each other as follows: one sibling pair (2); a father and two daughters (3); a mother, son and daughter (3); a mother and daughter (2). Control subjects were drawn from 27 siblings of the above patients who had genetically confirmed absence of 22q11.2DS or any other chromosomal abnormality and were closest in age to the case [13 males, 14 females; mean age 12.2 (SD 4.1). These cases and their sibling controls are a sub‐sample of a large, multinational study of individuals with 22q11.2DS [Schneider et al., 2014].

### 3D Laser Surface Imaging

**Figure 1:**
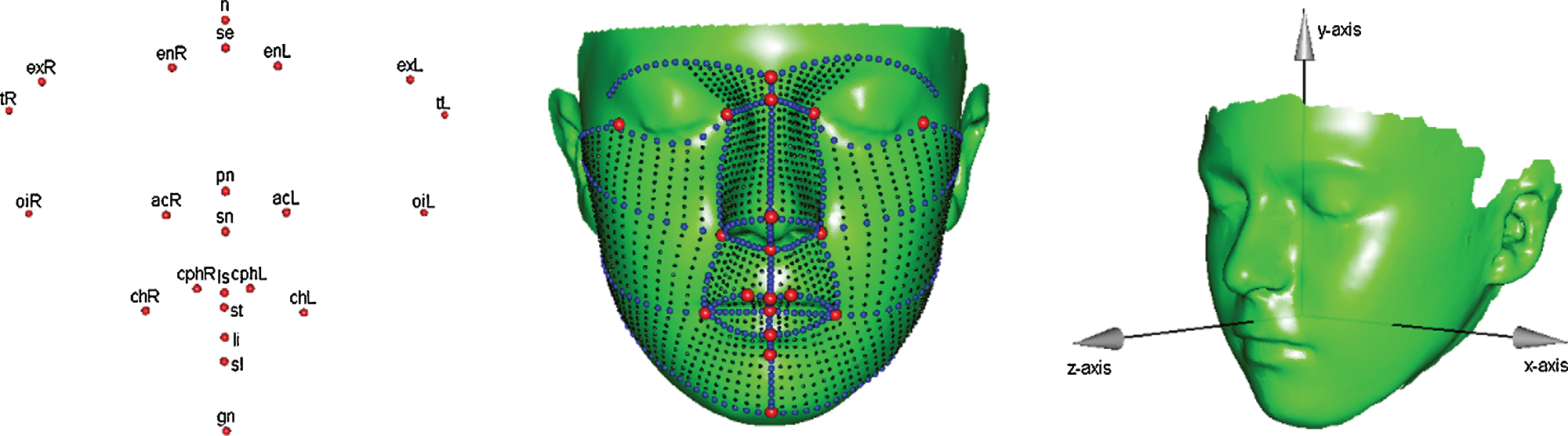
Mean facial shape across all subjects. Left, anatomical landmarks (red) and their abbreviated names with left side (L) and right side (R) in accordance with imaging conventions; Center, anatomical landmarks (red), semilandmarks on the curves (blue) and semilandmarks on the surface (black); Right, axis directions used in Figure 3.

Facial surfaces were recorded by a single investigator (SP) using a portable, hand‐held Polhemus FastScan laser scanner, as described previously [Hennessy et al., 2007, 2010]. A typical surface, consisting of ~80 000 points [~1,60,000 triangles], has been shown previously in detail [Hennessy et al., 2007] (Fig. 1).

### Facial Landmarks

Analysis proceeded on a paired, patient‐control basis. While the 27 unaffected sibling controls were those closest in age to the patient, siblings of the same sex were not always present in a given family; in those instances [n = 14], the control sibling was of the other sex, with statistical analysis controlling for effects of sex. After exclusion of 3D laser surface images for technical inadequacy (6 of 27 showed incomplete acquisition or patches where the surface had not been properly reconstructed), 21 patient‐control pairs were available for analysis (patients: 8 males, 13 females; mean age 11.0 (SD 3.8), range 6–19 years; controls: 10 males, 11 females; mean age 11.1 (SD 3.6), range 6–22 years; patient‐control pairs: 3 male‐male, 5 male‐female, 6 female‐female, 7 female‐male).

Craniofacial shape was characterized first by manually locating 30 biologically homologous anatomical landmarks (10 on the midline and 20 as right and left counterparts of each of 10 lateralized points). These landmarks, shown in Figure 1 and defined more specifically in Supplementary material I (Tables SI and SII), were identified by a single investigator (SK), who was blind to patient‐control status. This landmark set was augmented by 480 geometrically homologous semi‐landmarks (also known as pseudo‐ or interpolated landmarks) on curves and 1,720 on the surface to improve description of the face in regions where anatomical landmarks are not present. (Fig. 1) These semi‐landmarks were located by thin‐plate spline (TPS) warping [Bookstein, 1989] of a symmetric facial template onto each facial surface, using the anatomical landmarks as anchoring points [Hennessy et al., 2005]. The positions of the semi‐landmarks on each face were adjusted iteratively to create points that are geometrically homologous with respect to the template; this was achieved by minimizing bending energy between the template and each facial shape, which has the effect of removing artificial deformation [Bookstein, 1996].

### Geometric Morphometrics and Visualization

Generalized Procrustes analysis (GPA) [Dryden and Mardia, 1999] was used to match the entire set of faces by minimizing the Procrustes shape distance across location, orientation, and scale. This also allows a mean shape to be computed, a symmetrized version of which was used as a template for a second stage of iterative adjustment in order to improve accuracy. This process was repeated until convergence. For subsequent analysis, Procrustes shape co‐ordinates (PSC) were used, with the case‐control paired structure respected by analyzing differences and with adjustment for age and sex by a linear regression model; see Supplementary material II.

A shape‐space principal component analysis (PCA) [Hennessy et al., 2005; Bookstein, 1991] of patient‐control semi‐landmark differences was conducted; this multivariate model decomposes overall shape signal into low‐dimensional linear combinations of high‐dimensional measurements. In all cases, PCA was applied, as described in Supplementary material II, to seek and visualize differences between patient and control means. Statistical analyses were performed using the R software system [R Development Core Team, 2012]. For statistical tests, a permutation approach was adopted. This is a very useful method of performing an exact calculation when sample sizes are modest and/or exact distributional results are difficult to derive. It involves the comparison of observed case‐control differences with a set of differences derived from random permutations of the case‐control labels. These random permutations reflect a null hypothesis of no difference and so they provide a reference distribution against which the difference derived from the observed labeling can be compared. An empirical probability (*P*) value can be constructed simply from the proportion from the permutation sets that produce more extreme differences; 999 permutations were used.

## RESULTS

Among the 21 patient‐control pairs, PCA identified PCs 1–6 as explaining 75.2% of variance in overall shape space. Among these, PC2 distinguished patients and controls [*P* < 0.001] (Table I). In addition, PC6 [6.5% of variance] captured features that were asymmetric but did not differ between patients and controls [*P *= 0.99]; thus, they constitute subtle, intrinsic asymmetries of human facial shape in controls [Claes et al., 2012] that are unaltered in patients with 22q11.2DS.

**Table I:** Principal Component Analysis for Overall Shape Space

PC	Variance explained (%)	Cumulative variance (%)	*P*
PC1	25.3	25.3	.340
PC2	16.2	41.5	< .001
PC3	11.1	52.6	.065
PC4	8.1	60.8	.740
PC5	8.0	68.7	.870
PC6	6.5	75.2	.990

Variance explained by each principal component (PC), with permutation paired *t*‐test and associated *P* values for each PC in distinguishing patients from controls.

**Figure 2:**
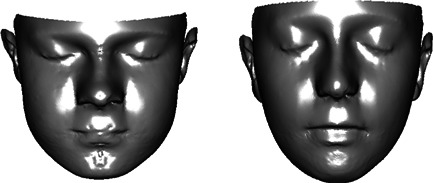
Visualization of PC2 as plain surfaces for (left) extreme control shape and (right) extreme patient shape.

**Figure 3:**
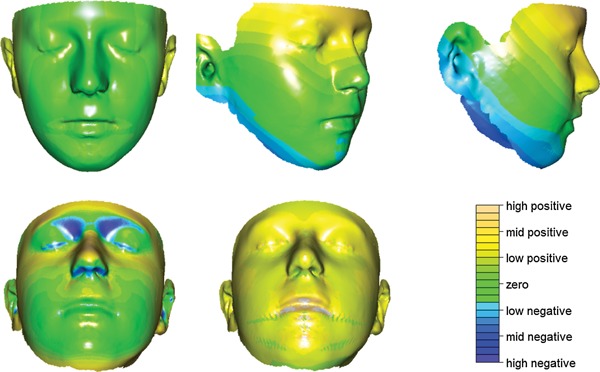
Visualization of PC2 as distances from extreme control shape to extreme patient shape at each point on the facial surface. Top row shows orthogonal components of 3D distances, with the same color scale used for all three directions: Left, differences along the *x*‐axis in coronal view; Center, differences along the *y*‐axis in coronal‐sagittal oblique view; Right, distances along the *z*‐axis in sagittal view. Bottom row: Left, 3D distances in the normal direction, i.e. perpendicular to the local surface area, in coronal‐transverse view; Center, patient‐control ratios for triangle areas in coronal‐transverse view; Right, color scale for 3D distances and triangle areas where positive [from mid‐green, through yellow to brown] indicates values for patients > controls and negative [from mid‐green, through blue to purple] indicates values for patients < controls.

**Figure 4:**
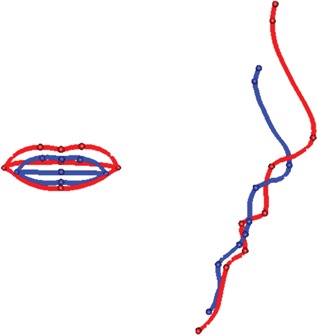
Visualization of PC2 as selected curves for extreme control (blue) and extreme patient (red) shapes. Left, upper lip, mid‐lip and lower lip curves in coronal view; Right, midline silhouettes from nasion to gnathion.

These statistical findings were given biological import through visualizations of PC2 by displaying images corresponding to the most extreme control and case shapes. Figure 2 shows the plain surfaces that correspond to extreme control shape and extreme case shape. Figure 3 shows the extreme case shape with added color to indicate the size of the change from control shape at each point on the surface. The top row of images represents movement in the *x*, *y*, and *z* directions of a face placed in an anatomical coordinate system with the line nasion–subnasale oriented vertically. Green‐toned colors indicate little movement, while blue‐toned reflect negative values and brown‐toned reflect positive values of the distances. The exception is the *x*‐direction, where movement is taken with respect to the mid‐line; thus, blue corresponds to narrowing and brown to widening. The images in the bottom row of Figure 3 show movement in the direction of the surface normal (left) and the case‐control ratio of the areas of the surface triangulation (middle). Figure 4 shows an alternative display for carefully chosen anatomical curves. In these images, cases are in red and controls in blue.

Using terminology for phenotypic variations that includes topographies from Elements of Morphology [Allanson et al., 2009a; Carey et al., 2012], these visualizations of PC2 indicate the following features of (i) head and face [Allanson et al., 2009b]; (ii) periorbital region [Hall et al., 2009]; (iii) nose and philtrum [Hennekam et al., 2009]; and (iv) lips, mouth and oral region [Carey et al., 2009] to statistically distinguish patients from controls.

### Head and Face

Face: long face, especially above the nasal tip (Fig. 3, top‐center). Forehead: prominent and slightly broad forehead up to the superior limit of acquisition (Fig. 3, top row); prominence of supraorbital ridges (Fig. 3, top‐right and bottom‐left). Maxilla and midface: prominence of midface with slight malar flattening that may reflect the more prominent lower forehead and mid‐face (Fig. 3, top‐right and bottom left); prominence of premaxilla (Fig. 3, bottom‐left; Fig. 4, right). Mandible: narrow jaw (narrow lower face; Fig. 3, top‐middle and top‐right); retrognathia/micrognathia (Fig. 3, top‐right; Fig. 4, right). There was a very slight increase in overall facial size in cases relative to controls (Fig. 3, bottom‐center).

### Periorbital Region

Upward and slightly lateral displacement of the eyes (Fig. 3, top‐middle); relative prominence of superolateral orbit (Fig.3, top‐right) but relative concavity of superomedial orbit (Fig. 3, bottom‐left); downslanting palpebral fissure with slight narrowing of eyelids (Fig. 2).

### Nose and Philtrum

Upward and slight forward displacement of the nose (Fig. 3, top‐right and bottom‐left; Fig. 4, right); increase in nasal length (Fig. 3, top‐center; Fig. 4, right); narrowing of the nasal root (Fig. 3, top‐right); prominence and roundness of the nasal tip (Fig. 3, top‐right; Fig. 4, right); narrowing of the nasal base (Fig. 2).

### Lips, Mouth and Oral Region

Prominence, thickness, and eversion of the vermilion (Fig. 3, bottom‐center; Fig. 4, left, right); open mouth posture (Fig. 2; Fig. 4, right); downslant of the mouth (Fig. 3, bottom‐center; Fig. 4, left).

## DISCUSSION

In this study we captured, analyzed and visualized over the whole facial surface abnormalities of 3D morphology in 22q11.2DS, with two objectives: First, to document, for the first time in its inherent 3‐dimensionality, the quantitative dysmorphology of 22q11.2DS craniofacies compared to sibling controls. The use of patient‐sibling controls is common in the study of 22q11.DS [Campbell et al., 2006; Howley et al., 2012], as it controls for family environment in relation to behavioral phenotype; family resemblance is not likely to be a major confounder, as this would favor similarities rather than differences between patients and their unaffected siblings. Second, to allow comparisons with the quantitative dysmorphology of schizophrenia.

Regarding 22q11.2DS, the present findings quantify and elaborate the facial characteristics described using classical clinical, anthropometric, and 2D photographic approaches, as recently reviewed [Butts, 2009]. Initial 3D surface imaging and geometric morphometric studies have described some congruent findings in clinically diagnosed 22q11.2DS compared to heterogeneous controls of unconfirmed genetic status; these descriptions related to analyses that focussed on the derivation of statistical models for diagnostic discrimination between subjects with Williams, Smith‐Magenis, 22q11.2DS and Noonan syndrome [Hammond et al., 2004, 2005].

In the present study, we supplemented geometric morphometrics of semi‐landmarks with construction and analysis of anatomical curves, geodesics, and surfaces to aid anatomical interpretation of visualizations of PC2, the shape space that here distinguished genetically confirmed 22q11.2DS from genetically confirmed sibling controls. Additionally, we included terminology for phenotypic variations based on topographies from Elements of Morphology for the standardization of human morphology [Allanson et al., 2009a; Carey et al., 2012]. Thus, our results reveal specific dysmorphology of the periorbital region, nose and philtrum, and lips, mouth and oral region, within more generalized dysmorphology of the head and face.

Patients with 22q11.2DS are at increased risk for psychosis to an extent exceeded only for monozygotic co‐twins of patients with schizophrenia [Murphy et al., 1999; Murphy, 2002; Bassett et al., 2005]; thus, comparisons of facial dysmorphology between these diagnostic groups has the potential to inform on shared and distinct aspects of developmental pathobiology. It must be taken into account that for schizophrenia the sexes were examined separately in patients and unrelated controls [Hennessy et al., 2007, 2010], while here, as previously [Hammond et al., 2004, 2005], in 22q11.2DS opposite sexes in some patient‐control sibling pairs required sex to be statistically removed from consideration; thus, comparisons are confined to those topographies of dysmorphology in schizophrenia that were most common to males and females.

The present findings were both, similar to and distinct from our previous 3D laser surface imaging and geometric morphometric studies in schizophrenia [Hennessy et al., 2007, 2010]: Findings were similar in terms of: overall widening of the upper face; lateral displacement of the eyes/orbits; prominence of the cheeks; narrowing of the lower face; narrowing of nasal prominences; posterior displacement of the chin. Findings were distinct in terms of: facial length (increased in 22q11.2DS (DS); decreased in schizophrenia (SZ)); mid‐face and nasal prominences (displaced upwards and outwards in DS; less prominent in SZ); lips (more prominent in DS; less prominent in SZ) and mouth (open mouth posture in DS; closed mouth posture in SZ).

Before discussing the biological import of these similarities and differences, it must be considered that while patients with schizophrenia have, by definition, manifested psychotic illness from young adulthood, those with 22q11.2DS include children who have not yet traversed the period of risk for psychosis and in whom sub‐threshold psychotic symptoms can be difficult to identify; no patient in the present study showed florid, psychotic symptoms.

Fundamental aspects of facial morphology are established early in development. However, some aspects are altered during the transition from childhood to adulthood. Increase in weight and overall facial size with age are not likely to be major confounders: age is taken into account in 22q11.2DS and schizophrenia analyses; importantly, similarities and differences in facial morphology between 22q11.2DS and schizophrenia were topographically specific, involving recessions/diminutions as well as prominences/expansions, in a manner inconsistent with an overall effect of age or weight.

Subjects with 22q11.2DS are commonly dichotomized into those who do and those who do not manifest psychotic illness subsequently. However, in reality the situation might be analogous to schizophrenia, where diagnosis may reflect the crossing of an arbitrary threshold along a dimension that extends from the breadth of psychotic ideation in the ‘normal’ population, through prodromal features (from brief, limited, intermittent psychotic symptoms and the putative attenuated psychosis syndrome), to clinical psychosis [Demjaha et al., 2009; van Os et al., 2009; Linscott and van Os, 2010; Waddington et al., 2012; Owoeye et al., 2013]; there is evidence that children/adolescents with 22q11.2DS show such sub‐clinical, prodromal features, the extent of which is associated with psychosis‐related psychopathological, cognitive and structural brain changes on a continuous rather than a dichotomous basis [Antshel et al., 2010; Kates et al., 2011; Armando et al., 2012; Schneider et al., 2012]. Thus, essentially all persons with 22q11.2DS share one or more risk factor(s) for psychosis that vary only in degree.

Therefore, the greater challenge is the extent to which the facial dysmorphology common to 22q11.2DS and schizophrenia may reflect a common pathobiological process associated with psychosis, while the facial dysmorphology that distinguishes 22q11.2DS from schizophrenia might reflect other, distinct pathobiological processes associated with distinct aspects of these disorders unrelated to psychosis. In recent molecular genetic studies, 22q11.2DS is recognized to be one of an increasing range of copy number variations (CNVs) associated with risk, not only for intellectual disability and psychosis, but also for autism spectrum disorder, attention deficit hyperactivity disorder, anxiety and depression, in association with congenital anomalies. In some individuals, 22q11.2DS may compound with a secondary CNV to result in a more severe clinical presentation [Kaminsky et al., 2011; Doherty et al., 2012; Girirajan et al., 2012].

At a cellular level, morphogenesis of the frontonasal prominences and forebrain are intimately regulated via epithelial‐mesenchymal signaling interactions: the nascent forebrain, neuroepithelium, neural crest and facial ectoderm, from which the present surface analyses derive, function as a developmental unit in terms of 3D gene expression domains, with maxillary and mandibular regions constituting distinct developmental domains [Diewert and Lozanoff, 1993 a, b; Diewert et al., 1993; Kjaer, 1995; Schneider et al., 2001; Echevarria et al., 2005; Marcucio et al., 2005, 2011; Tapadia et al., 2005; Minoux and Rijli, 2010; Szabo‐Rogers et al., 2010]. On this basis, the topography of craniofacial dysmorphology in schizophrenia and bipolar disorder [Hennessy et al., 2007, 2010] implicates events acting particularly over a time‐frame that has extreme limits of gestational weeks 6 through 19, with a common denominator of weeks 9/10 through 14/15 of gestation [Cohen et al., 1993; Diewert et al., 1993; Diewert and Lozanoff, 1993 a, b; Waddington et al., 1999 a, b; Bayer and Altman, 2005]. The dysmorphology evident here in 22q11.2DS overlaps with that in schizophrenia and bipolar disorder, suggesting dysmorphogenic events acting over a similar time‐frame. In contrast, dysmorphology evident in 22q11.2DS distinct from those in schizophrenia and bipolar disorder may indicate dysmorphogenic events (a) acting over a slightly differing time‐frame, (b) having a basis in the size of the 22q11.2 deletion, and/or (c) the involvement of a secondary CNV. These findings directly implicate dysmorphogenesis in a cerebral‐craniofacial domain that is common to 22q11.2 DS, schizophrenia and bipolar disorder and which may repay further clinical and genetic interrogation in relation to the developmental origins of psychotic illness.

## Supporting information

Additional supporting information may be found in the online version of this article at the publisher's web‐site.

Supporting Information.
