# Supplementary material for: Craniofacial dysmorphology in 22q11.2 deletion syndrome by 3D laser surface imaging and geometric morphometrics: Illuminating the developmental relationship to risk for psychosis
Source: Am J Med Genet A. 2015 Feb 18;167(3):529–36. doi: 10.1002/ajmg.a.36893 (PMC4737262; doi:10.1002/ajmg.a.36893)
Supplement: Supplementary file 1 — Supporting Information. [file AJMG-167-529-s001.doc]

SUPPLEMENTARY MATERIAL

Supplementary material I: Definitions of manually applied anatomical landmarks

**Table SI.** Craniofacial Landmarks.

| **Number** | **Label** | **Name (landmark category)** | **Locating profile** |
| --- | --- | --- | --- |
| **1** | prn | *pronasale* (hybrid 2/4) | Curvature |
| **2** | al | Right *alare* (4) | Curvature |
| **3** | al | Left *alare* (4) | Curvature |
| **4** | ac | Right *alare crest* (1) | Crossing of two curves |
| **5** | ac | Left *alare crest* (1) | Crossing of two curves |
| **6** | sn | *subnasale* (hybrid 2/4) | Curvature |
| **7** | nt | Right *nostril top point* (6) | Conventional |
| **8** | nt | Left *nostril top point* (6) | Conventional |
| **9** | cc | *columella* constructed point (6) | Conventional |
| **10** | nb | Right *nostril base point* (6) | Conventional |
| **11** | nb | Left *nostril base point* (6) | Conventional |
| **12** | se | *sellion* (4) | Curvature |
| **13** | N | *soft tissue* *nasion* (4) | Conventional |
| **14** | ek | Right *ektokonchion* (1) | Shape index |
| **15** | ek | Left *ektokonchion* (1) | Shape index |
| **16** | mf | Right *maxillofrontale* (1) | Shape index |
| **17** | mf | Left *maxillofrontale* (1) | Shape index |
| **18** | Ls | *labiale superius* (3b) | Conventional |
| **19** | cph | Right *crista philtri* (4) | Conventional |
| **20** | cph | Left *crista philtri* (4) | Conventional |
| **21** | ch | Right *chelion* (1) | Shape index |
| **22** | ch | Left *chelion* (1) | Shape index |
| **23** | sto | *stomion* (3b) | Conventional |
| **24** | Li | *labiale inferius* (3b) | Conventional |
| **25** | sl | *sublabiale* (hybrid 2/4) | Curvature |
| **26** | gn | *gnathion* (3b) | Crossing of two curves |
| **27** | T | Right *tragion* (hybrid 2/4) | Shape index |
| **28** | T | Left *tragion* (hybrid 2/4) | Shape index |
| **29** | oi | Right *otobasion inferius* (1) | Conventional |
| **30** | oi | Left *otobasion inferius* (1) | Conventional |

Landmarks can be assigned to six categories [Weber and Bookstein, 2011]:

- Type 1: discrete juxtaposition or intersection of tissues;
- Type 2: extremes of curvature (maximal or minimal) characterising a single structure;
- Type 3: characterized locally by information from multiple curves and surfaces and by symmetry:

- Type 3a: intersection of a ridge curve and the midsagittal curve on the same surface,

- Type 3b: intersection of an observed curve and the midsagittal curve,

- Type 3c: intersection of a ridge curve and an observed curve on the same surface;

- Type 4: semi-landmarks (see below) on ridge curves and symmetric curve (midsagittal curve);
- Type 5: semi-landmarks on surfaces;
- Type 6: constructed semi-landmarks.

In some instances, landmarks can be a mixture of types. The majority of landmarks are conventional [Farkas, 1994], and were located in Landmark software Wiley et al. [2005]. To aid location of some landmarks, 3D curvature of the particular curve was calculated [Koenderink and van Doorn, 1992]; thus, independence of facial orientation was achieved. Furthermore, some landmarks were identified also by local surface properties based on shape index [Koenderink, 1990], i.e. *cheilion* as spherical cup and *tragion* as saddle point. It was not possible to identify two of the traditional landmarks on 3D laser surface images due to closure of the eyes and absence of skin color, i.e. *exo-* and *endocanthion*; these were substituted by soft tissue *ektokonchion* (*ektoorbitale*) and soft tissue *maxillofrontale* (*anterior lacrimal point*). Definitions [Farkas, 1994], with the modifications below for those landmarks defined by shape index) include Type 2 landmarks [Bookstein, 1991] i.e. extremes of curvature by means of shape index (spherical cups, trough, rut, saddle rut, saddle, saddle ridge, ridge, dome or spherical cap points) that characterize a single location, as follows (Table SII):

**Table SII.** Definitions for Craniofacial Landmarks.

| **Number** | **Definitions by landmark category** |
| --- | --- |
| **1** | *pronasale* – most anterior midpoint of the nasal tip; midpoint of the nasal tip in the area of maximal local curvature, i.e. dome or spherical cap |
| **2/3 (L/R)** | *alare* – most lateral point on each alar contour |
| **4/5 (L/R)** | *alare* crest (*alare* curvature point) – point located at the facial insertion of each alar base, i.e. rut or saddle rut |
| **6** | *subnasale* – point at which the nasal septum merges with the upper cutaneous lip in the mid-sagittal plane; the point where the curvature (in the area of merging the nasal septum and the upper cutaneous lip) changes from positive to negative in the direction to the upper lip, i.e. saddle point |
| **7/8 (L/R)** | *nostril top point* (*columella* breakpoint) – highest point of each nostril or the superior terminal point of each nostril axis |
| **9** | *columella* constructed point – midpoint of the *columella* crest at the level of the nostril top points on the nasal septum |
| **10/11 (L/R)** | *nostril base point* – lowest point of each nostril or the inferior terminal point of each nostril axis |
| **12** | *sellion* – most posterior point of the frontonasal soft tissue contour in the midline of the base of the nasal root; point with minimal local curvature on the frontonasal soft tissue contour in the midline of the base of the nasal root, i.e. saddle ridge or ridge point |
| **13** | *soft tissue nasion* – midpoint on the soft tissue contour of the base of the nasal root at the level of the frontonasal suture |
| **14/15 (L/R)** | In our setting, *exocanthion* (defined as soft tissue point located at the outer commissure of each eye fissure) cannot be used due to closed eyes and is substituted by soft tissue *ektokonchion* (*ektoorbitale*) – perpendicular projection of hard tissue *ektokonchion* onto the skin, i.e. the point in the middle of lateral edge of the rim of eye socket, i.e. saddle rut or saddle |
| **16/17 (L/R)** | In our setting, *endocanthion* (defined as soft tissue point located at the inner commissure of each eye fissure) cannot be used due to closed eyes and is substituted by soft tissue *maxillofrontale* (anterior lacrimal point) – perpendicular projection of hard tissue *maxillofrontale* onto the skin, i.e. trough or spherical cup |
| **18** | *labiale superius* – midpoint of the vermilion line of the upper lip |
| **19/20 (L/R)** | *crista philtre* – point at each crossing of the vermilion line and the elevated margin of the philtrum |
| **21/22 (L/R)** | *cheilion* – point located at each labial commissure; the point of minimal local curvature, i.e. spherical cup |
| **23** | *stomion* – midpoint of the horizontal labial fissure |
| **24** | *labiale inferius* – midpoint of the vermilion line of the lower lip |
| **25** | *sublabiale* – most posterior midpoint on the labiomental soft tissue contour that defines the border between the lower lip and the chin; the midpoint of the area on labiomental soft tissue with minimal local curvature, i.e. rut |
| **26** | soft tissue *gnathion* – the most anterior-inferior midpoint of the chin; the midpoint of the chin with maximal local curvature, i.e. dome or spherical cap |
| **27/28 (L/R)** | *tragion* – point located at the upper margin of each tragius; the point of minimal local curvature at the upper margin of each tragius, i.e. saddle point |
| **29/30 (L/R)** | *otobasion inferius* – point of attachment of the ear lobe to the cheek, which determines the lower border of the ear insertion |

L, left; R, right.

**Supplementary material II: Geometric morphometrics**

The PSC were adjusted for age and sex by a linear regression model in the form

*centered PSC_ij_ = sex_i_ + age_i_ + sex_i_ : age_i_ + ε_ij_, i =1,2,..., 2230, j = 1,2,3,*

where *i* represents the (semi)-landmarks and *j* the dimension *x*, *y* or *z*. For further analysis of patients and controls, the residuals of this model were used.

In order to measure asymmetry [Mardia et al., 2000], the co-ordinates were relabelled and reflected (RR) with respect to the *yz*-plane, i.e. for paired anatomical landmarks, the sign and labels were reversed across the left-hand and right-hand side of the head shape. The PSC and their RR counterparts were jointly submitted to GPA to register all these shapes into the same space. Each PSC configuration was centered by subtraction of the mean of all PSC configurations and the same process was applied to the RR set. Fluctuating asymmetry expresses the difference between each PSC shape and its RR counterpart across the sample; it is calculated as the sum of squares of the Procrustes distances between each pair (asymmetry scores). The asymmetry of the means is calculated as the sum of squares of the Procrustes distances between the PSC and RR mean shapes. When multiplied by sample size, this is referred to as directional asymmetry. Differences in the directional asymmetry of cases and controls were analyzed by a paired *t*-test, using a permutation approach.

The PCA for reversible 3D images [Theobald et al., 2004] was performed for the 21 centred patient-control semi-landmark differences and their RR counterparts. This analysis allows symmetric and asymmetric PCs to be identified separately. The PC scores for each data point and its RR counterpart have the same absolute value. Symmetric PCs are those where the sign is also identical, while asymmetric PCs are those where the elements of the pairs have different signs. The position of each PC, in sequence of increasing variance explained, allows an interpretation of the contribution of asymmetry to variance. In considering patients *vs* controls, zero is the reference point of interest as this expresses the null model of no difference. Just as each PCA component projects the data onto a new scale, this null position was projected in the same manner, to provide a reference point on the new scale. Where this occurs, the nature of shape change from controls to patients can be identified through the change in PC score from the null position to the central point at zero, which corresponds to the mean patient-control difference. This gives a helpful interpretation to the direction of the PC. Differences in mean between patients and controls are then assessed by a permutation-based *t*-test on the PC scores.

Suitably scaled eigenvectors of the covariance matrix were added to the symmetrized mean (reference shape), calculated by RR of the mean shape semi-landmark coordinates. Using a carefully chosen mesh of 59,242 points triangulated by 117,386 faces and derived 2,200 semi-landmarks, we calculated standardized mesh points of the reference shape, i.e. TPS warp, by a TPS interpolation model. This process is performed in an anatomically and geometrically meaningful manner, similar to a dense correspondence model [Mao et al., 2006] where the sum of principal curvatures is equivalent to bending energies.

**References for Supplementary material**

Bookstein FL. 1991. Morphometric Tools for Landmark Data. New York: Cambridge University Press.

Farkas LG. 1994. Anthropometry of the Head and Face. New York: Raven Press.

Katina S, Bodorikova S, Dornhoferova M. 2012. Geometric and traditional morphometrics of human skull with respect to measurement reliability. Czech Anthropology 61:16-25.

Koenderink JJ. 1990. Solid Shape. Boston: MIT Press.

Koenderink JJ, van Doorn AJ. 1992. Surface shape and curvature scales. Image Vis Comput 10:557-564.

Mardia KV, Bookstein FL, Moreton IJ. 2000. Statistical assessment of bilateral symmetry of shapes. Biometrika 87:285-300.

Mao Z, Ju X, Siebert JP, Cockshott WP, Ayoub A. 2006. Constructing dense correspondence for the analysis of 3D facial morphology. Pattern Recog Letters 27:597-608.

Theobald CM, Glasbey CA, Horgan GW, Robinson CD. 2004. Principal components of landmarks from reversible images. Appl Stat 53:163-175.

Weber GW, Bookstein FL. 2011. Virtual Anthropology: A Guide to a New Interdisciplinary Field. New York: Springer.

Wiley DF, Amenta N, Alcantara DA, Ghosh D, Kil YJ, Delson E, Harcourt-Smith W, Rohlf FJ, St. John K, Hamann B, Motani R, Frost S, Rosenberger AL, Tallman L, Disotell T, O'Neill R. 2005. [Evolutionary morphing](http://ieeexplore.ieee.org/xpl/login.jsp?tp=&arnumber=1532826&url=http%3A%2F%2Fieeexplore.ieee.org%2Fxpls%2Fabs_all.jsp%3Farnumber%3D1532826). IEEE Transactions Vis Comput Graphics VIS 05:431-438 [see also <http://graphics.idav.ucdavis.edu/research/EvoMorph>]
